# Primary Multiple Pulmonary Primitive Neuroectodermal Tumor: Case Report and Literature Review

**DOI:** 10.1097/MD.0000000000001136

**Published:** 2015-07-13

**Authors:** Ming Dong, Jinghao Liu, Zuoqing Song, Xin Li, Tao Shi, Dan Wang, Dian Ren, Jun Chen

**Affiliations:** From the Department of Lung Cancer Surgery, Tianjin Lung Cancer Institute (MD, JL, ZS, XL, DR, JC); Department of Pathology; Tianjin Medical University General Hospital, Heping District, Tianjin, China (TS, DW).

## Abstract

Primitive neuroectodermal tumors (PNETs) arising directly from the lung are extremely rare but particularly aggressive neoplasms. Although thoracic PNET usually develops on the chest wall, there have been reports of primary Ewing sarcoma/PNET of the lung.

We present the case of a 16-year-old male with PNET diagnosed following histologic and immunohistochemical examination of a video-assisted thoracic surgical biopsy. As typically occurring for these tumors, the diagnosis was initially delayed in our patient and prognosis was poor despite aggressive surgical resection and postoperative chemotherapy. We attempted to explore the use of targeted pharmacotherapy through high-throughput sequencing in this case. We found that this treatment using Avastin and Cetuximab could provide a new therapeutic direction for PNET.

PNENTs arising from the lung parenchyma without pleural or chest wall involvement are extremely rare. Although uncommon, if the pathological features are similar to Ewing sarcoma, PNENTs should be kept in mind, and the target therapy may be a potent treatment for this disease.

## Abstract 2

Supplemental digital content is available in the text

## INTRODUCTION

Primitive neuroectodermal tumors (PNETs) belong to the Ewing sarcoma family, and most commonly arise in adolescents or young adults (usually younger than 35 years) with a slight male preponderance.^1^ PNET is a malignant neoplasm comprising small, undifferentiated neuroectodermal cells, and the common origin sites are the long bones, such as the femur and humerus as well as the pelvic bones. Reports have also noted the presence of PNET in the liver, kidneys, and adrenal glands.^2–4^ In the chest region, PNET commonly arises from the chest wall; this is usually referred to as an Askin tumor.^5,6^ Cases of PNETs arising from the lung parenchyma without pleural or chest wall involvement are extremely rare. To the best of our knowledge, multiple pulmonary PNTEs without chest wall involvement in 16-year-old adolescent have not been reported to date.

## PRESENTING CONCERNS

A 16-year-old boy was admitted with the chief complaints of shortness of breath and occasional chest pain of about 3 days duration. He had no other complaints and his past history was unremarkable. He denied smoking and had no family history of malignancy. A review of systems was noncontributory. Peripheral blood count, baseline serum chemistry screening, and urinalysis were normal on admission, as were tumor biomarker tests (alpha fetoprotein, serum ferritin, carcinoembryonic antigen, antigen 19-9, carbohydrate antigen 24-2, prostate-specific antigen, neuron-specific enolase, Cytokeratin 19 Fragment (CYFRA21-1), and squamous cell carcinoma antigen) and a purified protein derivative (PPD) for tuberculosis.

## CLINICAL FINDINGS

**FIGURE 1:**
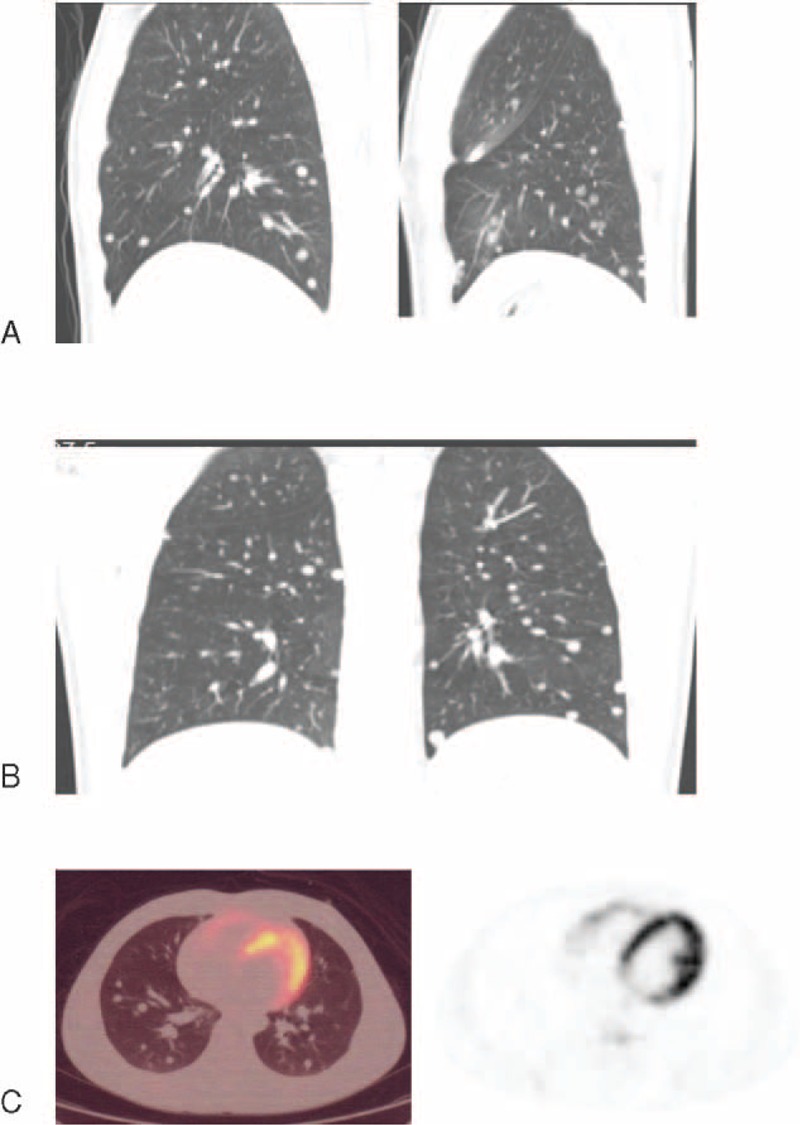
Chest CT scan shows multiple nodules in both lungs (A, B). PET-CT scan shows that there were no hypermetabolic lesions in either lung (C). CT = computed tomography, PET-CT = positron emission tomography-computed tomography.

As shown in Figure 1A, B, a computed tomography (CT) scan of the chest imaged multiple soft tissue nodules in both lungs, especially in both inferior lobes. The positron emission tomography-computed tomography (PET-CT) with fluorodeoxyglucose examination of total body revealed multiple soft tissue density nodules, with an approximate diameter 0.5 to 1.1 cm, in both lungs with a tracer concentration of SUV_max_ (standardized uptake value) of 1.0, and without correlative abnormality (Figure 1C). No other abnormal tracers were present. Magnetic resonance imaging of the brain was normal. Bronchoscopy was not performed.

## DIAGNOSTIC FOCUS AND ASSESSMENT

**FIGURE 2:**
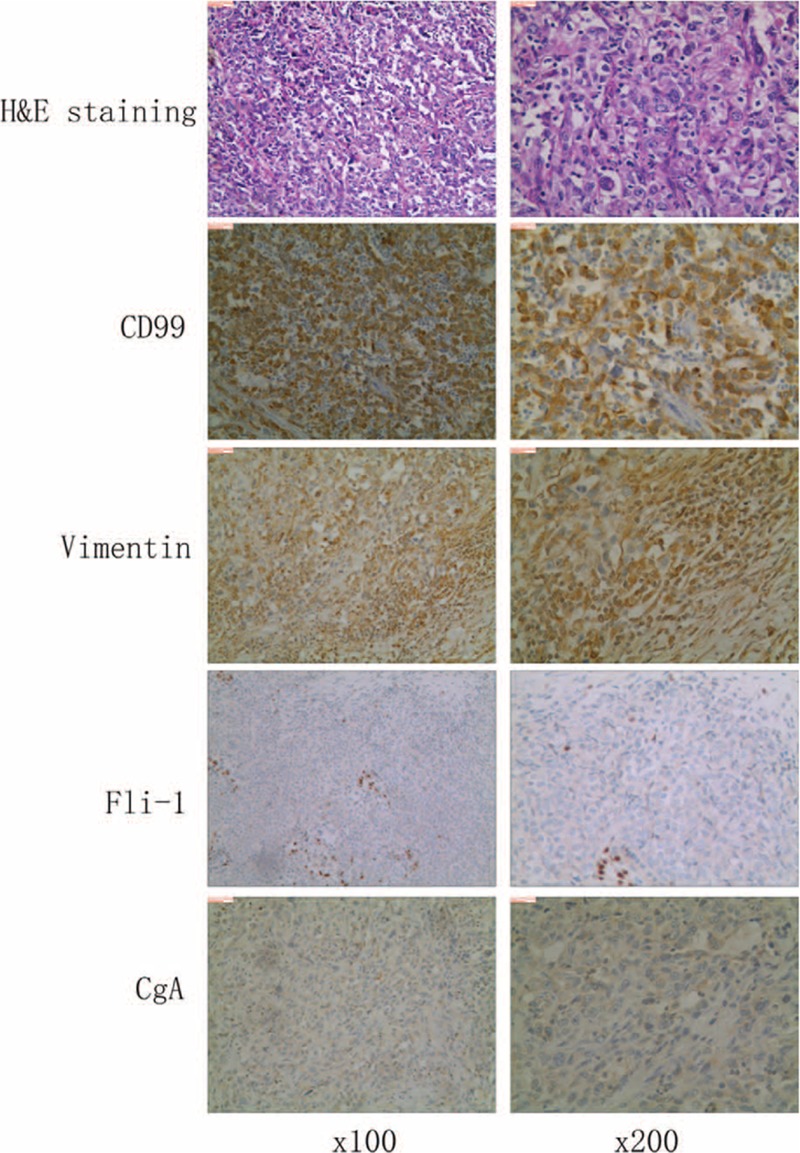
Histopathology. Hematoxylin–eosin (H&E) staining of the primary pulmonary primitive neuroectodermal tumor; immunohistochemistry staining of the primary tumor with different antibodies, such as anti-CD99, vimentin, chromogranin A (CgA), and friend leukemia integration 1 transcription factor (Fli-1). The images for the rest negative immunohistochemistry staining of thyroid transcription factor 1 (TTF-1), cytokeratin 7 (CK7), cytokeratin 19 (CK19), HMB45, protein S-100, epithelial membrane antigen (EMA), smooth muscle actin (SMA), CD34, CD20, CD56, CD31, leukocyte common antigen (LCA), and Desmin were not shown.

In order to establish the pathological diagnosis, a left-sided video-assisted thoracoscopy (VAT) was performed and 6 nodules in the lingual segment were removed. During the operation, a number of nodules were present on the surface of both left lung lobes with protrusion, but under the visceral pleura. As shown in Figure 2, histopathologic examination showed tumor cells that exhibited small, round, and mild morphology; partial cells with more cytoplasm; and partial cells with transparent cytoplasm. The majority of cells exhibited relatively consistent nuclear size. Immunostaining was positive for the expression of CD99 and vimentin and was negative for the expression of chromogranin A, thyroid transcription factor 1, cytokeratin 7, cytokeratin 19, human melanoma black 45 (HMB45), protein S-100, epithelial membrane antigen, smooth muscle actin (SMA), CD34, CD20, CD56, CD31, leukocyte common antigen, friend leukemia integration 1 transcription factor, and Desmin. In view of the foregoing, a diagnosis of primary lung PNET was made.

## THERAPEUTIC FOCUS AND ASSESSMENT

The patient had an uneventful postoperative recovery and was initially treated with the combined chemotherapy of Navelbine (25 mg/m^2^ day 1, 8), Cisplatin (80 mg/m^2^, day 1–2), and Endostatin (7.5 mg/m^2^, day 1–14), every 3 weeks at another facility. Since progressive disease was apparent after 2 cycles of this chemotherapy, the treatment regimen was changed to Ifosfamide (1.5 g/m^2^, day 1–5), Etoposide (100 mg/m^2^, day 1–3), and Endostatin (7.5 mg/m^2^, day1–14), every 3 weeks. After 1 cycle of this regimen, the disease continued to progress. Thus, the chemotherapy was changed to Paclitaxel (135 mg/m^2^, day1) and Gemcitabine (1 g/m^2^, day1, 8), every 3 weeks for 2 cycles. Although the chest CT scan did not image any obvious changes in the lung nodules, the patient complained of back pain, and multiple spinal metastases were imaged by magnetic resonance imaging.

## FOLLOW-UP AND OUTCOMES

Unfortunately, the patient expired 5 months after the initial diagnosis was made, as shown in Table 1 for the time course of his illness.

**TABLE 1:**
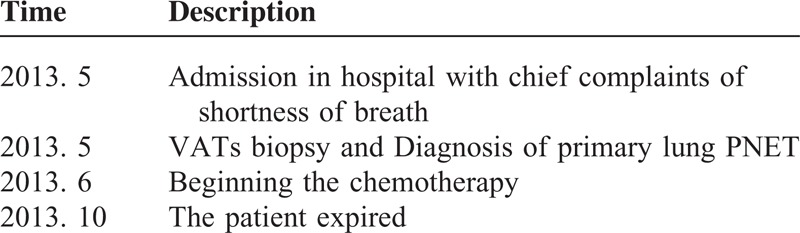
Timeline

Recently, in order to explore the molecular structure of the tumor, the mutations of 45 tumor-related driver genes were detected by a second-generation sequencing test (Beijing San Valley Biotechnology Inc., China). As shown in Complementary Table 1, http://links.lww.com/MD/A329, these driver genes include ALK, BRAF, EGFR, KIT, KRAS, PDGFRA, PIK3CA, ABL1, AKT1, APC, ATM, CDH1, CDKN2A, CSF1R, CTTNB1, ERBB2, ERBB4, FBXW7, FGFR1, FGFR2, FGFR3, FLT3, GNAS, HNF1A, HRAS, IDH1, JAK3, KDR, MET, MLH1, MPL, NOTCH1, NPM1, NRAS, PTEN, PTPN11, RB1, RET, SMAD4, SMARCB1, SMO, SRC, STK11, TP53, and VHL. In these genes, 737 known-mutation regions were detected and 9 mutations were present, which included 3 mutations occurring in known mutation regions and 6 mutations occurring in uncommon mutation regions. The primary database for the mutation genes were shown in Complementary Table 2, http://links.lww.com/MD/A329. These mutations include 8 genes: *HRAS*, *EGFR*, *MLH1*, *KIT*, *RET*, *FGFR3*, *APC*, and *PDGFRα*. Exception of *MLH1* gene with a missense mutation at encoding sequence 1151T>A indicated that the 384^th^ AA was changed from Val into Asp (p.384V[Val])→D[Asp]), the remaining 7 genes were all synonymous mutations, as shown in Table 2. These results indicated that there was nothing remarkable about these driver genes in this patient. Since there was a missense mutation of *MLH1* gene with the PNET in this patient, Avastin or Cetuximab could be used for the target therapy. However, this patient did not have the opportunity to receive this target therapy.

**TABLE 2:**
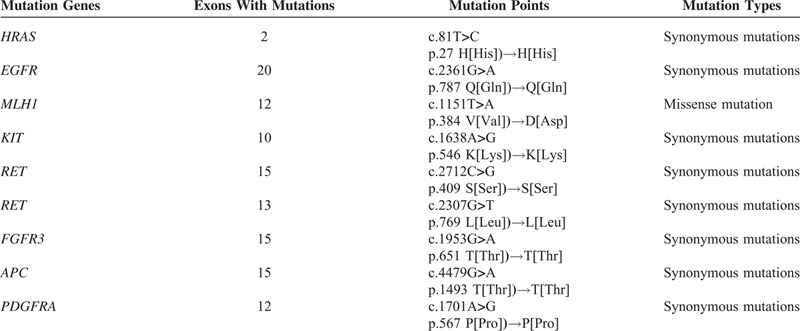
The Mutation Results of 8 Mutation Genes

Furthermore, we summarized the reported literatures for primary pulmonary primitive neuroectodermal tumor patients in Table 3. There were 20 cases reported in these literatures, which consisted with 12 males and 8 females. The age range was 8 to 75-years old with mean age of 30.6 years. In these 20 cases, there were 9 cases with the overall survival of 23.6 moths (from 5 to 54 moths), while there were still 9 cases alive with mean survival time over 17.2 months. Nineteen cases underwent resection plus neoadjuvant chemotherapy or adjuvant chemotherapy. These data suggested primary pulmonary PNETs often occur in young people with the age around 30-year, and the around 2-year overall survival is poor even with multiple treatments.

**TABLE 3:**
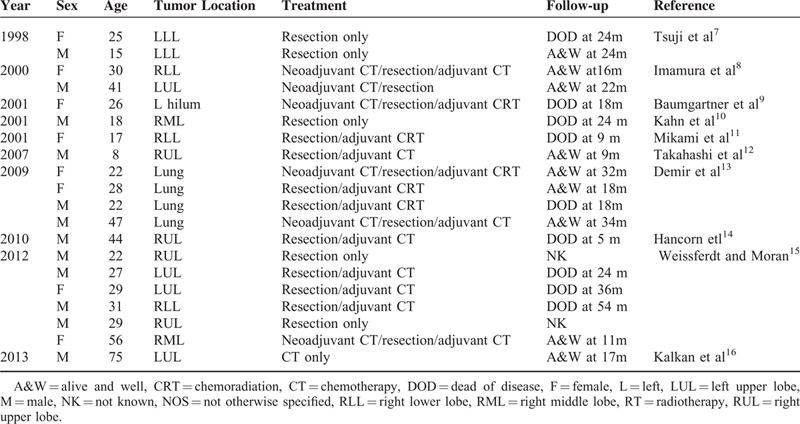
Primary Pulmonary Primitive Neuroectodermal Tumor Patients Reported in the Literatures

## DISCUSSION

PNETs and Ewing sarcoma are widely regarded as clinically and histologically identical tumors; they consist of small round cells and belong to the Ewing/PNET tumor family. They are uncommon entities and comprise 5% of all cases of small round cell tumors.^17^ But new research suggest that while the degree of neuronal differentiation used to be applied to distinguish between classical ES and PNET, molecular biology studies have now shown that all these tumors share a common gene rearrangement involving the *EWS* gene on chromosome 22, so that this distinction is now obsolete. In addition, PNETs are further divided into central and peripheral tumors. Peripheral primitive neuroectodermal tumors (pPNETs) occur outside the central nervous system and originates from neuroectodermal differentiation disorders.^18^ PNETs may affect people of all ages and occur most often in adolescents or young adults, usually before the age of 35 years with slight male preponderance.^1^ PNETs can occur in numerous solid organs including the kidneys, breasts, gastrointestinal tract, prostate, endometrium, jaw, adrenal glands, and meninges; however, they rarely arise in the lungs. In 1979, Askin et al^5^ described a similar rare malignant small-cell tumor of the thoracopulmonary region in 20 children and adolescents with a mean age of 14 years; they were referred to as Askin tumors. To the best of our knowledge, less than 20 cases of primary lung PNETs have been described in the literature. As shown in Table 3, a literature review for primary lung PNETs revealed that the average age of these patients was 30.6 (8–75) years, with a male preponderance (M:F = 1.5:1). The most common symptoms are cough, fever, dyspnea, hemoptysis, and chest pain, but no specific features. In addition, they usually produce swelling of the surrounding structures with some site-specific signs. Our case was a 16-year-old patient with symptoms consisting of chest pain and shortness of breath. The radiologic images and the video-assisted thoracoscopy procedure confirmed that the tumor originated from the lungs, but not from the chest wall or metastases from other locations. The diagnosis of this disease is primarily based on hematoxylin and eosin staining, which is characterized by monomorphic small round tumor cells, plus immunohistochemical (IHC) staining and cytogenetic analysis. Imaging tests such as CT scans exhibit heterogeneous masses, which often invade surrounding tissues, including bones. However, the images do not have specific features to distinguish the mass from other lung tumors. Histological evaluation and the utilization of some IHC markers and antibodies, such as O13, HBA-71, and 12E7 (the MIC2 gene product) to recognize the cell surface antigen, defined by the cluster of CD99, facilitate the diagnosis.^16^ Small cell carcinomas exhibit consistent positive immunoreactivity to cytokeratins, and reactivity for chromogranin and thyroid transcription factor 1. Malignant lymphomas do not absorb stain for leukocyte common antigen. Although there are no specific antibodies or markers for PNETs, CD99 is generally positive in these tumors. Our case also showed positive expression of CD99 by IHC staining, which was consistent with this point. In some cases, fluorescence in situ hybridisation or reverse transcription and polymerase chain reaction can also be used to confirm the diagnosis; However, IHC results are equivocal. A study reported that 85% of patients with these aggressive malignant tumors had the identification t(11;22)(q24;q12) chromosome rearrangement and the detection of p30/32 cell surface antigen (also known as the MIC2 gene product, which can be detected by antibodies such as HBA71 and O13), and the remaining 15% of the patients had variants of this translocation, including 22q12, 21q12 (10% of cases) and 7p22, 17q12, 2q36 (<1% of cases).^19^ Another study also noted that the structural changes might occur more often than translocations, including trisomy 8 or 12, deletions of 9p21.^20^ In our case, because the staining of CD99 was positive and the diagnosis of primary lung PNETs was confirmed, detection of the translocation was not conducted.

The treatment of PNET should be various combinations of early surgical resection as well as adjuvant chemotherapy and radiation therapy.^21^ As shown in a literature review,^16^ 8 of 20 (40%) patients underwent resection and adjuvant chemotherapy with or without radiation, while 36/20 (30%) patients underwent neoadjuvant chemotherapy followed by resection with or without adjuvant chemotherapy, 5/20 (25%) patients underwent resection, and 1/20 (5%) patient only received chemotherapy. The 2-year survival rates for the first three groups were 37%, 33%, and 60%, respectively, and the patient who only underwent chemotherapy lived for 17 months following the initial diagnosis. To the best of our knowledge, our case is the first to report a patient with inoperable thoracic PNET who only underwent the chemotherapy, and lived only 5 months after the initial diagnosis was made. Ewing tumor family is generally comprised of PNETS, Askin tumors (Ewing sarcomas affecting the chest wall), and extraosseous Ewing sarcoma. Due to the significant progression in understanding disease pathogenesis and multimodality treatments, 1-year survival rate of patients with localized Ewing sarcoma has increased up to nearly 70%. However, the 5-year survival rate is still very poor, less than 25% approximately.^22^ PNET, like other Ewing sarcomas, is a highly malignant tumor with poor prognosis. Once the diagnosis is made, multimodality treatments, including systemic chemotherapy (neoadjuvant and adjuvant), surgery, and radiation, are all considered as treatment alternatives depending on the disease status and patients condition.

The first-line drugs of proven efficacy for Ewing sarcoma include vincristine, doxorubicin, cyclophosphamide, etoposide, and ifosfamide.^23^ For the patients with metastatic disease, the second-line regimens, including cyclophosphamide/topotecan, irinotecan/temozolomide, and high-dose ifosfamide, are often exploited.^24,25^ So far, there was no evidence comparing the therapeutic outcomes of surgery and radiotherapy for the localized Ewing sarcoma. Therefore, based on the clinical experience, surgical resection is the preferential alternative, followed with radiotherapy with a dosage <40 Gy for the positive margin.^26^

Targeted therapy is also an effective therapeutic alternative in Ewing sarcoma, and some potential biomarkers have been addressed.^27,28^ Previous studies have demonstrated that angiogenesis played an important role in the growth of Ewing sarcoma. In the COG-AEWS-0521 trial, it was found that the patients with recurrent Ewing sarcoma exhibited a high level of VEGF protein. And animal studies also proved a role for angiogenesis in Ewing sarcoma.^24,29,30^ Therefore, bevacizumab (Avastin) and sunitinib might be the good candidates of targeted therapy for Ewing sarcoma. In our case, the missense mutation of the *MLH1* gene indicated that Avastin or Cetuximab was a possible targeted therapy alternative. However, it was unfortunate that this patient was not received these medications. Moreover, Ecteinascidin 743 (Trabectedin) has been used for the treatment of soft tissue sarcoma, but the effect still needs to be determined for Ewing sarcoma.^31^

In view of the foregoing, the outcome of PNETs is very poor due to the limitations of traditional chemotherapy and radiotherapy. Furthermore, to date, the target therapy for this disease remains unproven. To the best of our knowledge, the literature contains no other similar cases with the formation of multiple nodules in both lungs. Our case highlights the outcome of detecting the mutations of oncogenes. Our results suggest that we could attempt a target medicine cure using Avastin and Cetuximab. Moreover, it might provide a new therapeutic direction.

## PATIENT CONSENT

The patient signed the permission for the publication when he was admitted in our hospital at the first time.
